# Regulation of α-bergamotene biosynthesis by the LcDOF5.8-LcTPSbms regulatory module in litchi fruit

**DOI:** 10.1016/j.fmre.2025.12.004

**Published:** 2025-12-17

**Authors:** Yimeng Wang, Zhuoyi Liu, Minghui Wang, Dawei Qian, Ke Ma, Minglei Zhao, Jianguo Li, Xingshuai Ma

**Affiliations:** a Key Laboratory of Biology and Genetic Improvement of Horticultural Crops (South China), Ministry of Agriculture and Rural Affairs, Guangdong Litchi Engineering Research Center, College of Horticulture, South China Agricultural University, Guangzhou 510642, China; b Dongguan Agricultural Science Research Center, Dongguan 523086, China

**Keywords:** Fruit aroma, TPS, Terpenes, Sesquiterpenes, Bergamotene, DOF, Transcription factor, Litchi

## Abstract

Bergamotene, a key sesquiterpene compound, is crucial for defining the fruity aroma. However, the precise regulatory mechanisms governing its biosynthesis have been largely elusive. In this study, we elucidated that the DOF transcription factor LcDOF5.8 plays a central role in the biosynthesis of α-bergamotene by directly enhancing the expression of the terpene synthase gene *LcTPSbms* in litchi fruit. Our findings revealed that the concentration of α-bergamotene varies significantly among four distinct litchi cultivars: ‘Guanyinlv’, ‘Bingli’, ‘Guiwei’ and ‘Nuomici’. Notably, a strong correlation was observed between the expression level of *LcTPSbms* and the content level of α-bergamotene across these cultivars. Furthermore, both *in vitro* and *in vivo* catalytic assays confirmed that LcTPSbms is capable of catalyzing the synthesis of α-bergamotene. Importantly, electrophoretic mobility shift assays (EMSA) and Dual-LUC assays demonstrated that *LcTPSbms* is positively regulated by LcDOF5.8. Silencing *LcDOF5.8* in litchi aril resulted in decreased *LcTPSbms* expression, whereas transient overexpression of *LcDOF5.8* led to a significant upregulation of *LcTPSbms* and a concomitant increase in α-bergamotene biosynthesis. In summary, our research uncovers a regulatory module involving LcDOF5.8 and LcTPSbms that plays a critical role in the biosynthesis of α-bergamotene, offering valuable insights into the molecular mechanisms underlying the fruity aroma of litchi fruit.

## Graphical abstract

**Figure:**
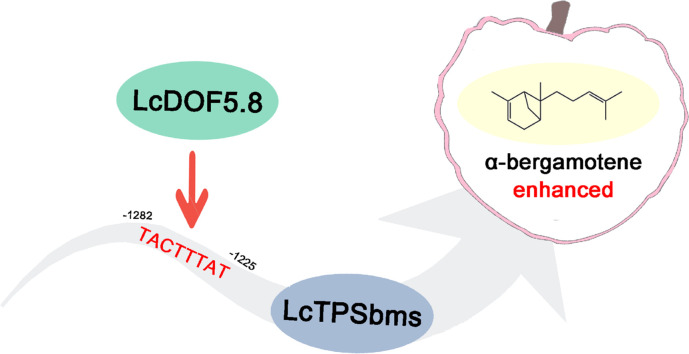

## 1 Introduction

The fruit aroma, a pivotal characteristic of horticultural plants, is intricately woven from the interplay of terpenes, phenylpropanes, and fatty acid derivatives. The distinct content and proportion of these compounds create the variety-specific aroma [[1], [2], [3], [4]]. Terpenes, in particular, stand out as the most extensive and diverse class of chemical compounds found across all living organisms [5]. Economically, terpenes' significance spans horticulture, perfumery, therapeutics, and flavorings [6]. Derived from C_5_ isoprene units, terpenes exhibit remarkable structural diversity [7]. Up to 2017, >80,000 terpenes have been identified [8]. This underscores the complexity and richness of the terpenoids, which significantly contribute to the unique and captivating aroma of horticultural fruit.

Terpenes encompass a diverse range, primarily comprising monoterpenes (C_10_), sesquiterpenes (C_15_), and diterpenes (C_20_) [9]. Among these, sesquiterpenes form the largest subgroup, boasting extensive applications in pharmaceuticals, biofuels, flavors, and fragrances [10,11]. Notably, bergamotenes, a member of sesquiterpenes, have garnered significant attention in recent years. Bergamotenes, as constituents of plant essential oils, are predominantly found in bergamot, cannabis, lime, and basil [[12], [13], [14]]. Among them, α-bergamotene and β-bergamotene are two crucial structural isomers, differing solely in the position of a double bond [15]. Predominantly occurring, α-bergamotene exudes a sweet and citrusy aroma, making it a popular choice in perfumery, food, and skin care products [[16], [17], [18], [19]]. Furthermore, bergamotenes have demonstrated the antioxidant, anti-inflammatory [20], anti-feedant [21], immunosuppressive [22], and anticancer properties [23], opening up avenues for potential applications in pharmaceuticals, nutraceuticals, cosmeceuticals, and pest management [24]. However, despite these promising findings, the underlying regulatory molecular mechanism of bergamotene biosynthesis remains largely elusive.

Terpene synthases (TPSs), a well-characterized family of enzymes, serve as metabolic sentinels, orchestrating the biosynthesis of an array of terpene compounds [[25], [26], [27], [28], [29]]. It has been well established that the expression level of *TPS* genes exhibits a tight correlation with the content of terpenes. In *Freesia*, for instance, the preferential expression patterns of *FhTPS1* in petals and pistils, and *FhTPS4* in calyxes and receptacles, coincide precisely with the differential distribution of linalool [30]. Similarly, in tree peony, the cultivar-specific emission of linalool is determined by the expression profiles of *PdTPS1* and *PdTPS4* across different cultivars [31]. Thus far, several TPSs have been pinpointed for their role in the production of bergamotene across various species. For instance, in sweet pea, LoTPS7 specifically catalyzes the formation of α-bergamotene [32]. In maize, the overexpression of *TPS10* triggers the enhanced production of α-bergamotene alongside other sesquiterpenes [33]. Furthermore, the recombinant enzyme SaSSy in sandalwood has been shown to prominently generate α-bergamotene from (Z, Z)-farnesyl diphosphate [34]. Similarly, in sorghum, both SbTPS3 and SbTPS5 possess the capability to synthesize α-bergamotene [35]. However, the transcriptional regulation of these *TPSs* remains largely unexplored.

Previous studies have highlighted the intricate interplay of various factors that influence the *TPS* gene expression. Transcription factors (TFs) have been identified as key regulators in the intricate network of factors that orchestrate *TPS* gene expression. To date, MYB, bZIP, NAC, bHLH, WRKY, and AP2/ERF TFs have been validated as regulators of *TPS* genes [36,37]. For instance, MdMYC2 and MdERF3 positively co-activated the expression of *MdAFS* and increased the production of farnesene in apple [38]; In peach, the transcription factor PpERF61 has been shown to regulate the expression of *PpTPS1* and *PpTPS3*, which are responsible for linalool synthesis, and could be regulated by [39]; In maize, ZmEREB58 activates *TPS10*, and then influences the production of β-farnesene and α-bergamotene [40]. Among the myriad TFs, DOFs represent a unique class of plant-specific factors that execute diverse functions throughout the plant’s life cycle, including roles in hormone signaling, responses to abiotic stress, light perception, carbon and nitrogen metabolism, and seed germination [41]. Currently, the potential role of DOFs in volatile terpene biosynthesis remains to be fully explored. Whether DOFs can regulate the expression of *TPS* genes needs further verification.

Litchi (*Litchi chinensis* Sonn.), a fruit crop cherished for its tropical and subtropical cultivation over two millennia, is renowned for its complex bouquet of volatile terpene compounds [42]. Prior studies have detected a total of 556 volatile terpene compounds in litchi, with the content and composition of various monoterpenes and sesquiterpenes being pivotal to its rose-like or citrus-like aroma [43]. Among these, sesquiterpenes such as bergamotenes are particularly significant, yet the specific LcTPSs involved in their biosynthesis and the molecular mechanisms regulating this process have been largely enigmatic. In our current investigation, we observed that the expression of *LcTPSbms* was strongly correlated with the level of α-bergamotene. Further, the expression of *LcTPSbms* was found to be directly enhanced by the transcription factor LcDOF5.8. The transient overexpression of *LcDOF5.8* in litchi aril led to an increase in the production of α-bergamotene, underscoring its regulatory influence. Collectively, our findings uncover a novel regulatory module involving LcDOF5.8 and LcTPSbms that governs the formation of α-bergamotene.

## 2 Materials and methods

### 2.1 Plant materials

Four distinct litchi cultivars with diverse flavors: ‘Guanyinlv’ (GYL), ‘Bingli’ (BL), ‘Guiwei’ (GW), and ‘Nuomici’ (NMC) were meticulously selected from the Dongguan Botanical Garden in Guangdong, China. These cultivars were grown under uniform conditions. Litchi fruits were harvested at 70, 77, and 84 days after pollination (DAP) from three representative trees of each cultivar. Each tree served as an independent biological replicate, and samples from each tree were composed of a mixture of 25 fruits with consistent growth conditions. Immediately after collection, the samples were snap-frozen in liquid nitrogen to preserve their metabolic integrity and stored at −80 °C for subsequent experiments.

### 2.2 Gas chromatography-mass spectrometry (GC-MS) analysis

In a 20 mL headspace vial, 500 mg of litchi aril was combined with 2 mL of saturated NaCl solution and 0.5 µg of an internal standard. The samples were then subjected to an incubation process at 60 °C with gentle shaking for 5 min. Following this, a 120 µm DVB/CWR/PDMS fiber from Agilent was introduced into the vial and allowed to adsorb volatile compounds for 15 min. Subsequently, the captured volatile compounds were desorbed and transferred to an Agilent 8890-7000D GC-MS/MS system, which was equipped with a DB-5MS capillary column (30 m in length, 0.25 mm in diameter, and a 0.25 µm film thickness) for analysis. Identification of the volatile compounds was achieved by comparing their mass spectra against the NIST 2011 mass spectra library. Finally, the content of the volatile terpene compounds was quantitatively determined using a specified formula:

the content of volatile terpene compounds (µg/kg · FW) = target peak area × 0.5 ÷ internal standard peak area ÷ sample weight (kg)

### 2.3 RNA extraction and sequencing

Total RNA was extracted from litchi aril using the Column Plant RNAout kit provided by TIANGEN, Beijing. Following extraction, the transcriptome sequencing was performed by Biomarker Technologies Co., Ltd, utilizing the Illumina platform. Before data analysis, reads with low quality, the proportion of *N* > 5%, and connector contamination were removed to ensure the reliability of the results. The sequence data were filtered to remove adapters using the Trimmomatic software [44]. The transcriptome sequencing data were then analyzed using the BMKCloud platform (www.biocloud.net) and Tbtools [45].

### 2.4 Weighted correlation network analysis

Weighted Gene Co-expression Network Analysis (WGCNA) [46] was performed using the WGCNA shiny plugin by TBtools [45] with default parameters, apart from the soft threshold power of 10, module cuttree height was 0.6 and min module size was 100.

### 2.5 Quantitative real-time PCR

To synthesize the first-strand cDNA, 1 µg of RNA was processed in accordance with the instructions of the TransScript One-Step gDNA Removal and cDNA Synthesis SuperMix Kit (TransGen, Beijing). Quantitative Real-time PCR reactions were subsequently conducted on an ABI7500 Quantitative Real-time PCR System from Applied Biosystems, employing SYBR Green PCR Supermix from Bio-Rad. Each Quantitative Real-time PCR analysis was replicated three times to ensure reliability. The expression level of the tested genes was quantitatively determined using the formula 2^-△△Ct^, with *EF-1α* serving as the actin gene as established by Zhong *et al*. [47].

### 2.6 Subcellular localization analysis

The coding sequences of *LcDOF5.8* were subcloned into the pEAQ-GFP vector, incorporating a GFP reporter, with the specific primers detailed in Table S1. Subsequently, *Agrobacterium tumefaciens* strain GV3101 cells, each harboring either pEAQ-LcDOF5.8-GFP or the GFP control vector, were individually transfected into tobacco leaves (*Nicotiana benthamiana*). After 56 h of infiltration, the GFP signals were visualized using a confocal laser scanning microscope (LSM 7 DUO, ZEISS, Germany) at a wavelength of 488 nm.

### 2.7 Heterologous expression of *LcTPSbms* protein in *E. coli* and *in vitro* enzyme assay

The *LcTPSbms* gene was amplified and subsequently seamlessly subcloned into the pMAL-c2X-MBP vector. The LcTPSbm*s*-MBP recombinant protein was purified utilizing MBPSep Dextrin Agarose Resin (Yeasen, Shanghai). Following established protocols [32,48], 100 µg purified protein was combined with either 2 mM GPP (G6772) or FPP (F6892) (Sigma, Germany) as substrate, along with an assay buffer comprising 7.5 mM MgCl_2_, 3.3 mM KCl, 0.6 mM MnCl_2_, 5 mM dithiothreitol (DTT), and 5% (v/v) glycerol in a 50–90 mM HEPES solution adjusted to pH 7.4. The mixtures were incubated at 30 °C for 2 h, and subsequently, the volatile compounds were analyzed by GC-MS. As a negative control, an empty pMAL-c2X-MBP vector was employed under identical conditions.

### 2.8 *In vivo* characterization of *LcTPSbms*

The pEAQ-LcTPSbms-GFP plasmid was successfully transformed into *Agrobacterium tumefaciens* strain GV3101 cells, which were then utilized to infiltrate the leaves of 4-week-old *Nicotiana benthamiana* plants. During the infiltration process, a 1:1 ratio of *Arabidopsis thaliana* GPP synthase 1 (AtGDS1, NP 850, 234.1) or FPP synthase 2 (AtFPS2, NP 001328530.1) was co-injected to facilitate the enzymatic reactions. Four days post-infiltration, 10 infected leaves were collected and mixed. Then, 2 g of the sample was weighed out and placed in a transparent, sealed container for subsequent analysis of volatile compounds. As a negative control, an empty pEAQ-HT-GFP vector with AtGDS1 or AtFPS2 (at a 1:1 ratio) was utilized under identical conditions.

### 2.9 Electrophoretic mobility shift assay (EMSA)

The coding sequence of *LcDOF5.8* was amplified and subsequently cloned into the pGEX-4T-1-GST vector, resulting in the expression of the fusion protein LcDOF5.8-GST. This fusion protein was subsequently purified using GSTSep Glutathione Agarose Resin (YEASEN, Shanghai). The probes utilized in the assays were designed to include the DOF binding site (TACTTTAT) derived from the *LcTPSbms* promoter, and were biotin-labeled at their 3′ end. As competitors, identical sequences without biotin labeling and a mutant probe in which the DOF binding site (TACTTTAT) was altered to CGTCCCGC were employed. The LightShift Chemiluminescent EMSA Kit (Thermo Scientific, Illinois, USA) was chosen to conduct the electrophoretic mobility shift assays (EMSAs). The images were captured using the ChemiDoc MP Imaging System (Bio-Rad, Hercules, CA, USA).

### 2.10 Yeast one-hybrid assay

The yeast one-hybrid assay was performed according to the method described by Ma *et al*. [49]. For prey vector construction, the coding sequence of *LcDOF5.8* was amplified and inserted into the pGADT7 vector. For bait vector construction, the *LcTPSbms* promoter fragment was cloned into the pAbAi vector. Subsequently, the yeast one-hybrid assay was conducted using the Matchmaker™ Gold Yeast One-Hybrid Library Screening System (BD Clontech, Palo Alto, CA, USA). DNA-protein interactions were assessed based on the growth of transformed yeast on SD/-Leu medium containing Aureobasidin A.

### 2.11 Dual-luciferase reporter assay

To ascertain the transcriptional activity of LcDOF5.8, we utilized the dual-luciferase transient expression system. Specifically, the coding sequence of *LcDOF5.8* was cloned into the pGreen II 62-SK vector, serving as the effector. Meanwhile, the promoter region of *LcTPSbms*, which harbors the DOF binding sites, was fused into the pGreen II 0800 vector and designated as the reporter. In accordance with our previously published protocol [50], the effector and reporter constructs were transiently co-transformed into tobacco leaves. Following an infiltration period of 48∼72 h, the activities of both firefly luciferase (LUC) and renilla luciferase (REN) were measured using the Dual-luciferase assay reagents (YEASEN, Shanghai). The ratio of LUC to REN was then calculated to quantify the transcriptional activity of LcDOF5.8. This experimental procedure was replicated at least six times to ensure consistency and reliability.

### 2.12 Transient overexpression of *LcDOF5.8* in litchi aril

As described in a previous study [51], the aril of the litchi was first disinfected and subsequently cut into small fragments. These fragments were then transformed with the *Agrobacterium tumefaciens* strain GV3101, which harbored the *LcDOF5.8* gene, under a vacuum pressure of 70 kPa. Following the vacuum infiltration process, the litchi aril was thoroughly rinsed with sterile water three times. Subsequently, the aril was cultured on murashige and skoog (MS) medium for an initial period of 2 days in darkness, followed by 3∼5 days under normal light conditions. As a negative control, *Agrobacterium tumefaciens* carrying an empty vector was utilized in parallel.

### 2.13 Virus-induced gene silencing of *LcDOF5.8* in litchi aril

A 297 bp fragment of *LcDOF5.8* (LITCHI011105: 19 bp∼315 bp) was cloned and inserted into the pTRV RNA2 (pTRV2) vector to generate the pTRV-LcDOF5.8 construct. The recombinant construct was transformed into *A. tumefaciens* strain GV3101. Mixtures of cultures containing equal pTRV RNA1 (pTRV1) and pTRV2-LcDOF5.8 construct were transformed into litchi airl as described above. Samples were collected after 7 days of transformation for further detection.

### 2.14 Data analysis

All experimental data presented herein were expressed as the mean value derived from either three or six independent biological replicates, ensuring robust statistical representation. Statistical significance was rigorously assessed using either the Student’s *t*-test or Duncan’s test, depending on the specific requirement of the analysis. Furthermore, the primers employed in this study are comprehensively listed in Table S1.

## 3 Results

### 3.1 The content of α-bergamotene varies among litchi cultivars

**Fig. 1:**
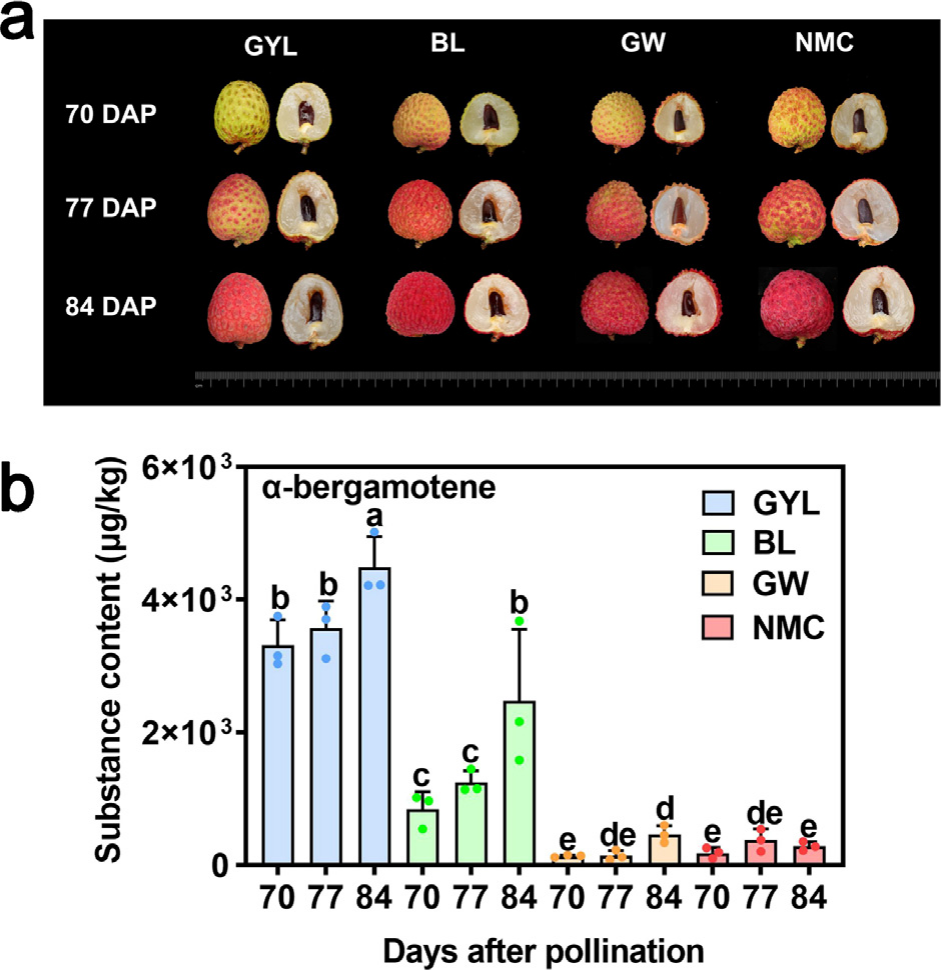
**α-bergamotene content in the aril of four litchi cultivars.** (a) Images of three fruit development stages of ‘Guanyinlv’ (GYL), ‘Bingli’ (BL), ‘Guiwei’ (GW), and ‘Nuomici’ (NMC) litchi cultivars. At 70 days after pollination (DAP), the litchi fruit is in the color-changing period; At 77 DAP, the fruit tends to be mature, and the fruit reached full maturation at 84 DAP. (b) The content of α-bergamotene at 70, 77, and 84 DAP in ‘GYL’, ‘BL’, ‘GW’, and ‘NMC’ litchi cultivars. Different letters indicate significant pairwise differences according to the Duncan’s test (*p* < 0.05).

Fruit samples were collected at 70, 77, and 84 days after pollination (DAP), corresponding to key stages in the litchi's development. At 70 DAP, the litchi fruit experiences a color shift, nearing maturity by 77 DAP, and reaching full maturity by 84 DAP (Fig. 1a). Post-harvest, we conducted an analysis of the bergamotene content within the aril of four litchi cultivars at the specified DAP stages. Evidently (Fig. 1b), α-bergamotene was detectable in all four cultivars. Generally, the content of α-bergamotene exhibited a progressive increase across the three developmental stages in the four litchi cultivars, mirroring the fruit maturation process. Notably, there were variations in the α-bergamotene content among the cultivars at the same developmental stage. Specifically, ‘GYL’ had the highest α-bergamotene content, followed by ‘BL’. In contrast, ‘NMC’ showed the lowest level, closely trailed by ‘GW’ at full maturity (84 DAP). In summary, these findings indicate that the content of α-bergamotene varies significantly among litchi cultivars, highlighting the diversity of this aromatic compound across different varieties. This variation underscores the potential for targeted breeding and cultivation practices to enhance the aroma profile of litchi fruit.

### 3.2 The expression level of *LcTPSbms* is highly correlated with the content of α-bergamotene in litchi aril

**Fig. 2:**
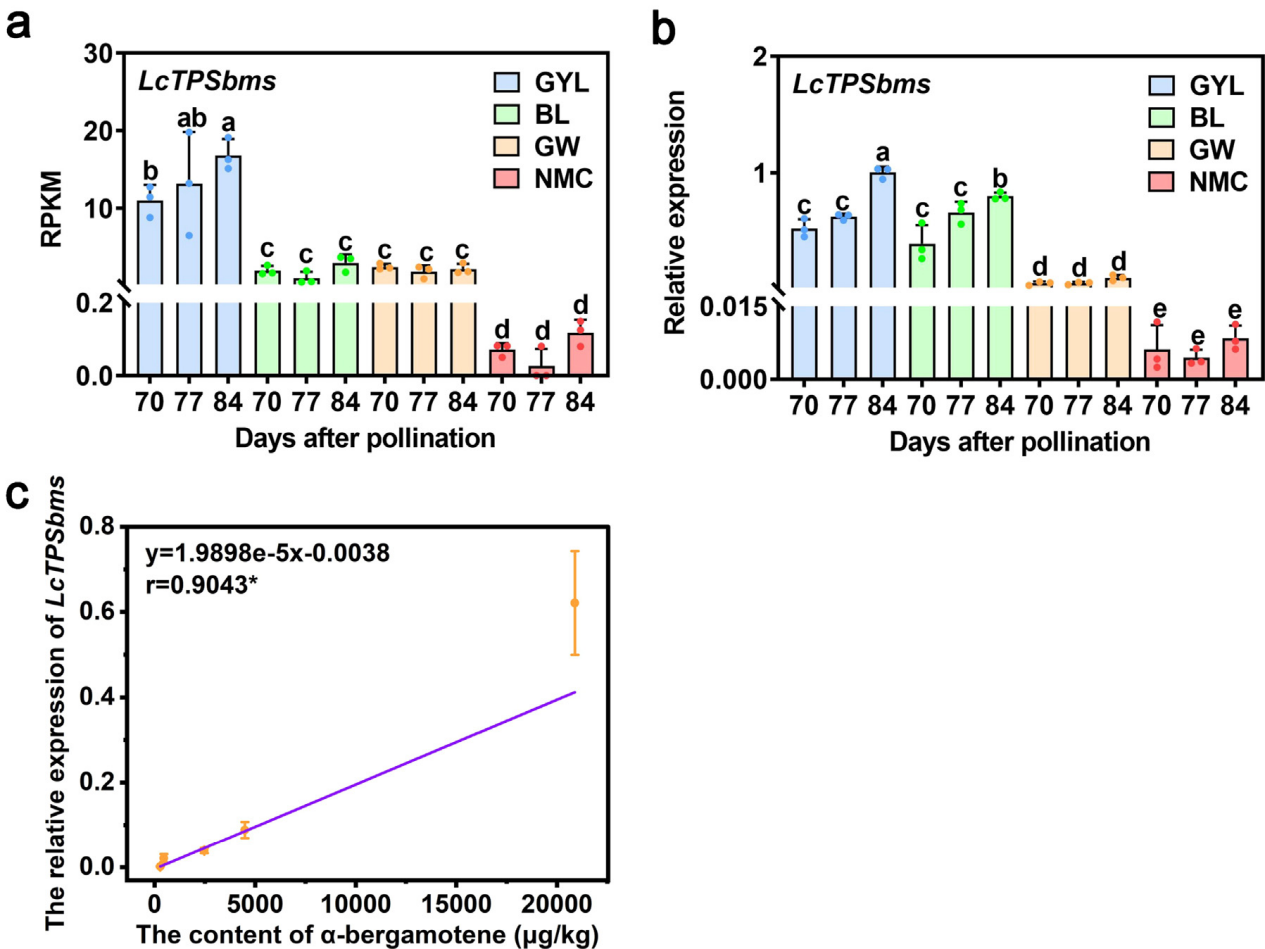
**The expression patterns of *LcTPSbms* in ‘GYL’, ‘BL’, ‘GW’, and ‘NMC’ litchi cultivars.** The expression levels of *LcTPSbms* at 70, 77, and 84 DAP in ‘GYL’, ‘BL’, ‘GW’, and ‘NMC’ litchi cultivars under RNA-seq (a) and qRT-PCR (b) analysis. RPKM: Reads Per Kilo base per Million reads. (c) Correlations between the content of α-bergamotene and the expression level of *LcTPSbms* at 84 DAP in ‘GYL’, ‘BL’, ‘GW’, and ‘NMC’ litchi cultivars. The error bars indicate mean ± SD. Different letters indicate significant pairwise differences according to the Duncan’s test (*p* < 0.05). At least three independent experiments were performed.

To identify the specific *LcTPSs* involved in the synthesis of α-bergamotene within the litchi aril. We detected the content of α-bergamotene in four litchi cultivars among three developmental stages. Further, to screen the *TPSs* gene responsible for the synthesis of α-bergamotene, we employed RNA sequencing (RNA-seq) and quantitative real-time PCR (qRT-PCR) technologies. Through RNA sequencing (RNA-seq) and quantitative real-time PCR (qRT-PCR), we identified a novel *LcTPS* gene (LITCHI002932, designated as *LcTPSbms*, Fig. S1) within the litchi genome. Based on the RNA-seq result, *LcTPSbms* exhibited a high level of expression that increased progressively in the ‘GYL’ cultivar, with ‘BL’ and ‘GW’ cultivars showing similar trends. In contrast, the ‘NMC’ cultivar displayed significantly lower *LcTPSbms* expression (Fig. 2a). However, there were minor inconsistencies between the RNA-seq and qRT-PCR results (Fig. 2b), which were more consistent with the α-bergamotene content across the four litchi cultivars at three developmental stages. Notably, the expression of *LcTPSbms* differed significantly among the four cultivars at 84 DAP. The ‘GYL’ cultivar had the highest *LcTPSbms* expression, closely followed by ‘BL’. In contrast, ‘NMC’ exhibited the lowest level, with ‘GW’ showing a slightly higher level. Importantly, *LcTPSbms* expression peaked at 84 DAP across all three developmental stages within each cultivar. At maturity (84 DAP), the expression of *LcTPSbms* in ‘GYL’ and ‘BL’ was significantly higher than in the previous two stages, while no significant differences were found in ‘GW’ and ‘NMC’. Furthermore, a correlation analysis revealed a strong relationship between *LcTPSbms* expression levels and α-bergamotene content across the ‘GYL’, ‘BL’, ‘GW’, and ‘NMC’ cultivars (*r* = 0.90), as shown in Fig. 2c. Collectively, these findings suggest that *LcTPSbms* expression is highly correlated with α-bergamotene content, suggesting a role for *LcTPSbms* in regulating the biosynthesis of α-bergamotene in litchi aril.

### 3.3 The catalytic activity of *LcTPSbms*

It is well-established that monoterpenes are synthesized within plastids, where GPP is formed, whereas sesquiterpenes are synthesized in the cytoplasm utilizing FPP as a precursor [[52], [53], [54]]. To ascertain the functional role of LcTPSbms, we analyzed the *in vitro* enzyme assay and the *in vivo* characterization.

**Fig. 3:**
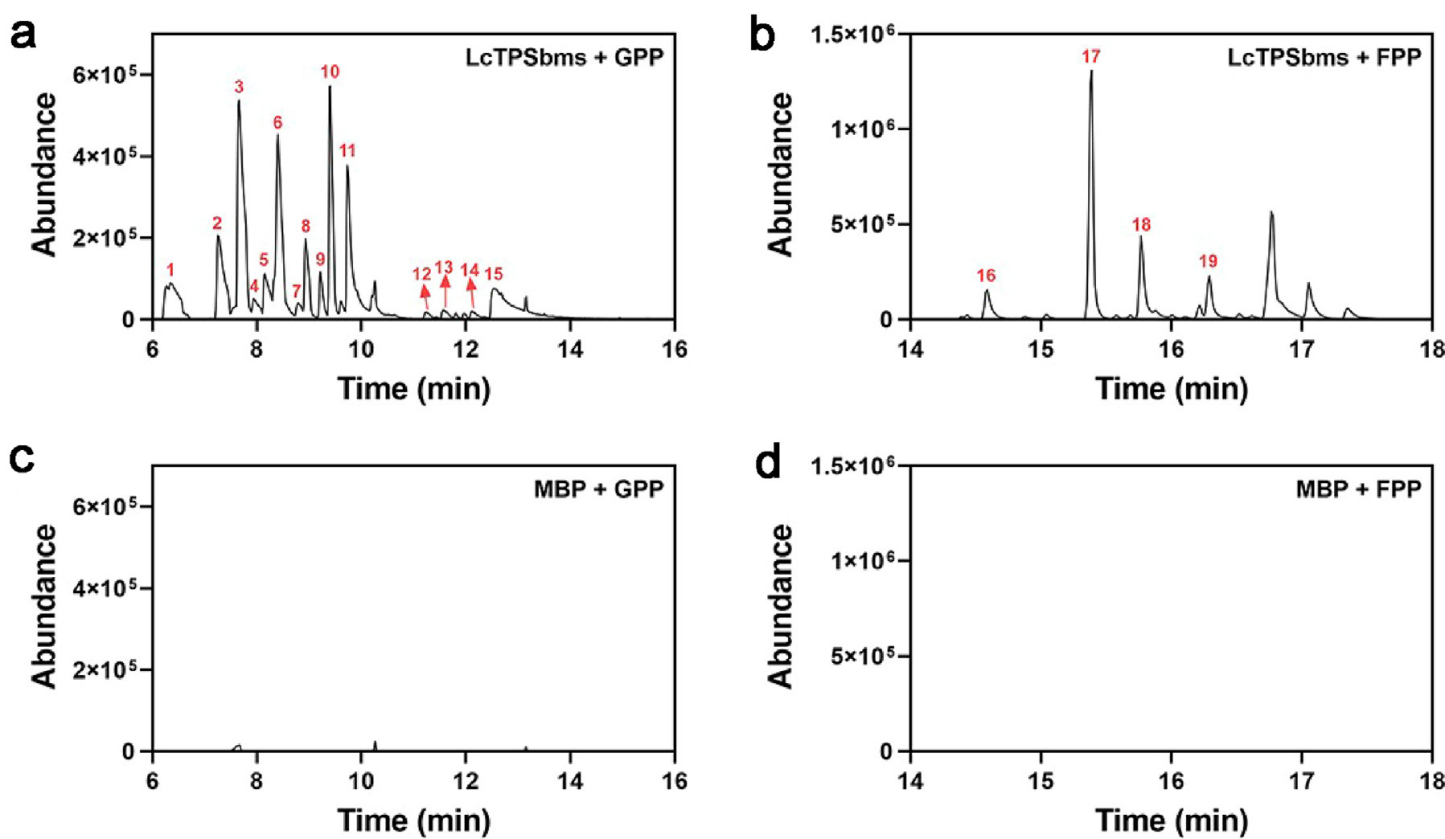
***In vitro* assays of the catalytic activity of *LcTPSbms*.** Enzymatic analysis of *LcTPSbms* using GPP (a) or FPP (b) as substrance. The *LcTPSbms*-MBP purified protein was combined with substrate. The mixtures were incubated at 30 °C for 2 h, and then the volatile compounds were analyzed using GC-MS. Compounds 1 to 22 represent different volatiles: 1. α-thujene 2. sabinene 3. myrcene 4. α-phellandrene 5. α-terpinene 6. β-phellandrene 7. β-ocimene 8. γ-terpinene 9. trans-β-terpineol 10. 4-carene 11. linalool 12. terpinen-4-ol 13. α-terpineol 14. nerol 15. geraniol 16. α-zingiberene 17. α-bergamotene 18. β-farnesene 19. α-curcumene. The empty pMAL-c2X-MBP protein was employed as negative control to combine with GPP (c) or FPP (d), respectively.

To validate the catalytic activity of LcTPSbms, the recombinant LcTPSbms-MBP protein was first purified. Subsequently, *in vitro* catalytic assays were conducted using both GPP and FPP as substrates. When GPP was utilized, LcTPSbms-MBP catalyzed the formation of 15 distinct monoterpenes, including myrcene (20.92%), β-phellandrene (17.17%), 4-carene (12.92%), linalool (10.13%), sabinene (9.63%), α-terpinene (5.82%), γ-terpinene (5.54%), geraniol (3.78%), trans-β-terpineol (2.59%), α-thujene (2.02%), decamethylcyclopentasiloxane (1.52%), α-phellandrene (1.35%), ocimene (0.95%), α-terpineol (0.83%), and nerolidol (0.73%) (Fig. 3a). Conversely, when FPP served as the substrate, the primary products were sesquiterpenes, specifically α-bergamotene (31.58%), β-farnesene (12.00%), α-zingiberene (5.99%) and α-curcumene (0.31%) (Fig. 3b). Notably, no volatile compounds were detected when MBP protein alone was incubated with either GPP or FPP (Fig. 3c, d), indicating the specific catalytic activity of LcTPSbms-MBP.

**Fig. 4:**
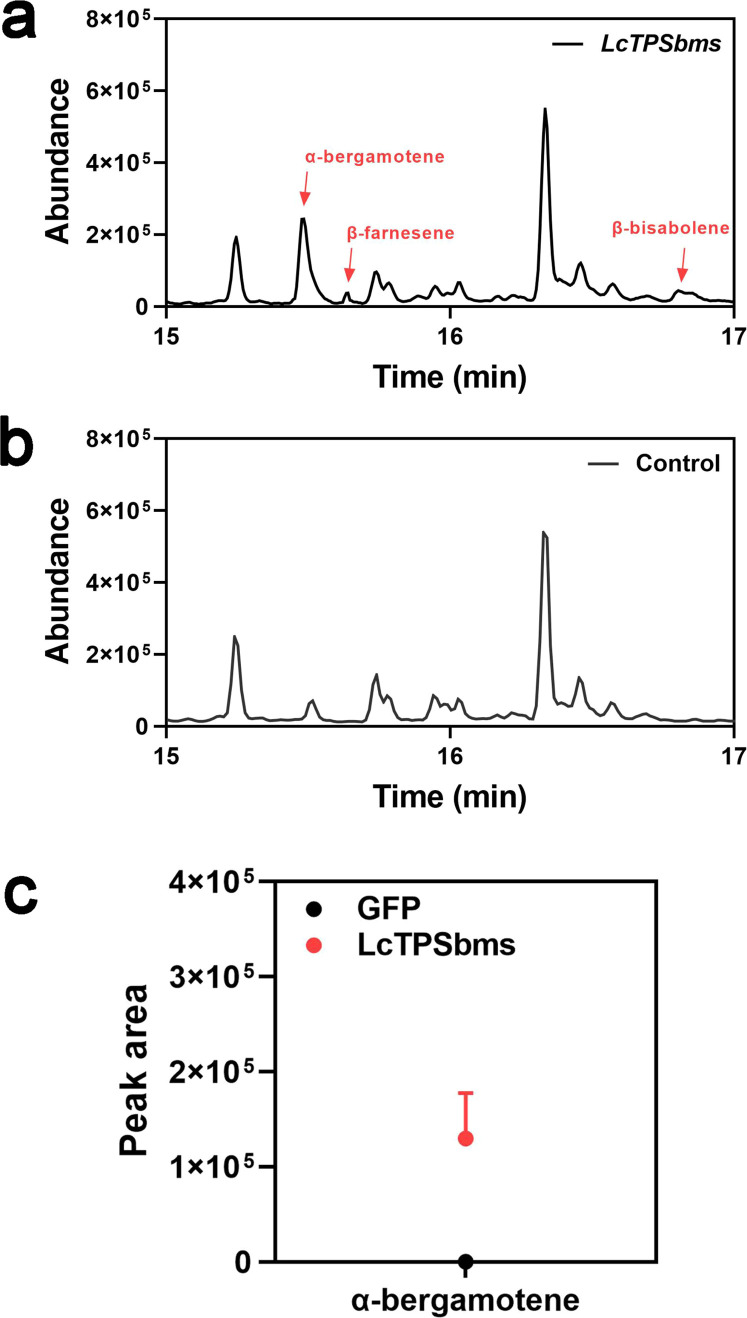
***In vivo* assays of the catalytic activity of *LcTPSbms*.** The pEAQ-*LcTPSbms*-GFP (a) and pEAQ-GFP control (b) were transiently overexpressed into tobacco leaves, respectively. After 4 days infiltration, the volatile compounds were detected utilizing GC-MS. The α-bergamotene, β-farnesene, and β-bisabolene compounds were indicated in red. The peak area of detected α-bergamotene was analyzed (c).

To further confirm the functional property of LcTPSbms, we transiently overexpressed *LcTPSbms* in *Nicotiana benthamiana* leaves and subsequently performed GC-MS analysis to investigate the volatile terpene compounds. For illustration (Fig. 4a), compared to the control (Fig. 4b), the overexpression of *LcTPSbms* in tobacco leaves resulted in a notable increase in the production of specific sesquiterpenes. Three prominent sesquiterpenes were identified: α-bergamotene (6.81%), β-bisabolene (0.96%), and β-farnesene (0.30%). The enhancement in the α-bergamotene content, catalyzed by LcTPSbms, was particularly significant compared to the control, as quantified in Fig. 4c. These observations underscore the capacity of LcTPSbms to catalyze the biosynthesis of these specific sesquiterpenes in litchi, suggesting its pivotal role in the aroma formation of the fruit.

Taking into account the *in vitro* and *in vivo* enzymatic findings, it is reasonable to conclude that the significance of LcTPSbms in the metabolic pathway leading to the production of α-bergamotene in litchi.

### 3.4 Identification of candidate TFs that are co-expressed with *LcTPSbms*

**Fig. 5:**
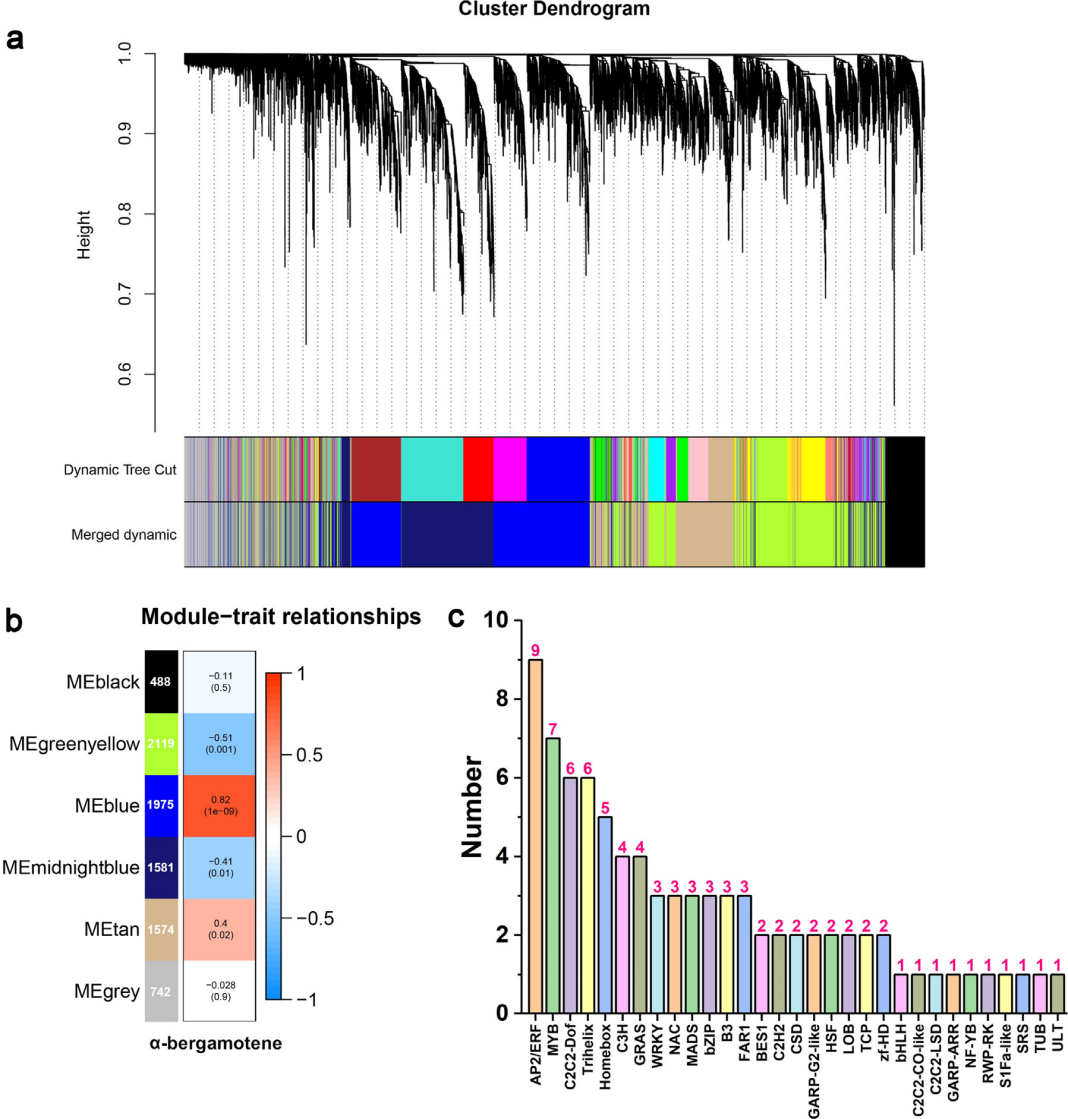
**Identification the key transcription factors involved in the biosynthesis of α-bergamotene by weighted gene co-expression network analysis (WGCNA).** (a) Clustering tree of differentially expressed genes based on topological overlap matrix (TOM). (b) Correlation analysis of module with the content of α-bergamotene in ‘GYL’, ‘BL’, ‘GW’, and ‘NMC’ litchi cultivars at 70, 77, and 84 DAP. (c) Classification of 85 transcription factors related to the content distribution of α-bergamotene in ‘GYL’, ‘BL’, ‘GW’, and ‘NMC’ litchi cultivars at 70, 77, and 84 DAP.

Our current investigation delves into the differential expression of *LcTPSbms* across three developmental stages in four litchi cultivars, which correlates closely with the content distribution of α-bergamotene (Fig. 2). This correlation suggests that upstream regulatory elements may play a crucial role in modulating *LcTPSbms* expression and the biosynthesis of α-bergamotene among these cultivars. To comprehensively pinpoint the key TFs regulating the expression of *LcTPSbms* and the biosynthesis of α-bergamotene, a weighted gene co-expression network analysis (WGCNA) was performed. This analysis revealed a total of six distinct co-expression modules (Fig. 5a), with the gene count within each module detailed in Fig. 5b Subsequently, a correlation analysis was undertaken between the identified gene modules and the α-bergamotene content across the four litchi cultivars at three developmental stages. The results (depicted in Fig. 5b) indicated that the blue module exhibited the strongest correlation with α-bergamotene content distribution, notably encompassing the *LcTPSbms* gene. Consequently, genes residing within the blue module were further analyzed to identify potential regulators of *LcTPSbms*. As shown in Fig. 5c, transcription factor prediction analysis of the blue module revealed a total of 85 TFs, predominantly belonging to the AP2/ERF (eight members), MYB (seven members), C2C2-Dof (six members), Trihelix (six members), and Homebox (five members) families. These TFs are likely to be intricately linked to both the expression of *LcTPSbms* and α-bergamotene biosynthesis.

### 3.5 *LcDOF5.8* activates the expression of *LcTPSbms* by directly binding to its promoter

To further elucidate the transcriptional regulation mechanism governing *LcTPSbms*, we conducted a comprehensive analysis of its promoter region utilizing the JASPAR^2024^ website and PlantCARE resources. As a result, we identified the DOF transcription factor as the candidate upstream regulator of *LcTPSbms*. As there are six DOF TFs co-expressed with *LcTPSbms* (Fig. 5c), we further analyzed the expression patterns of these six DOF TFs using data from the *Sapindaceae* genome database (http://www.sapindaceae.com/index.html). As illustrated in Fig. S4, *LcDOF5.8* exhibited the highest expression level in the aril. Based on these results, LcDOF5.8 was selected as the key regulator of *LcTPSbms* for further analysis.

**Fig. 6:**
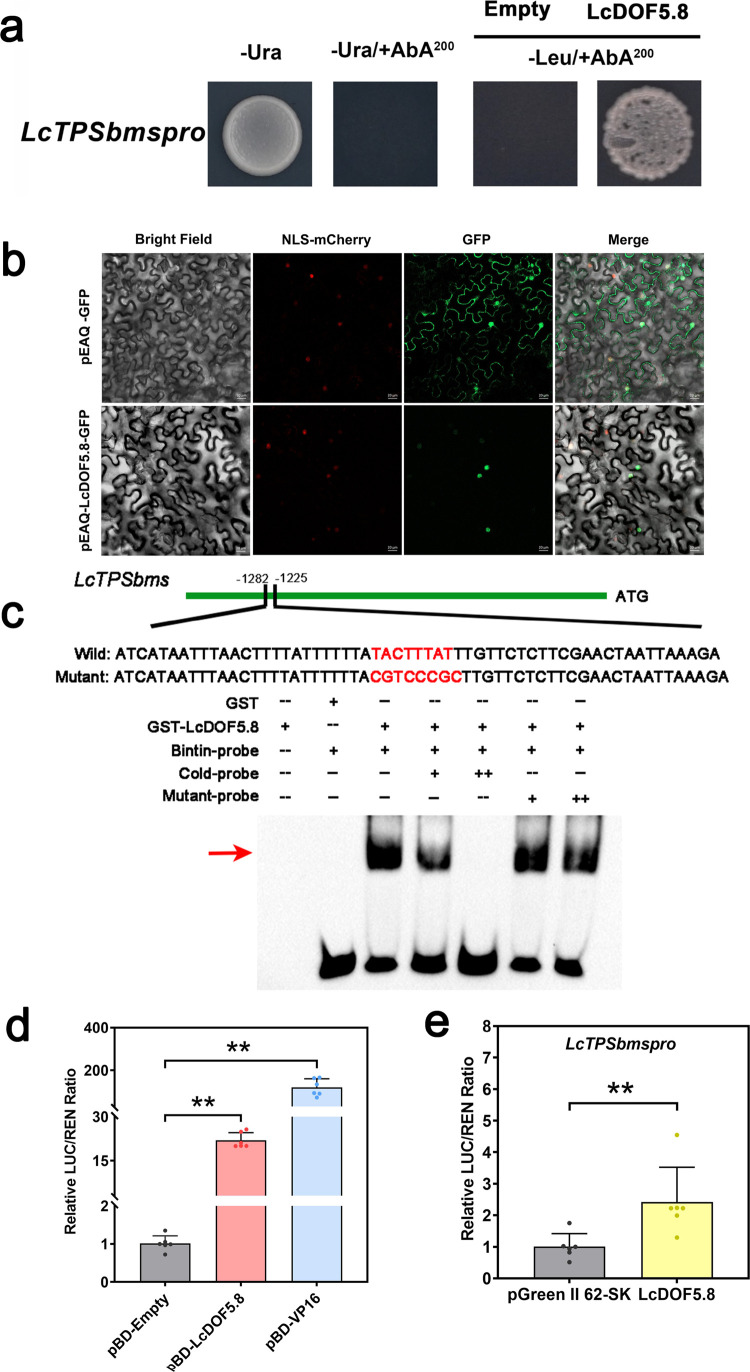
***LcDOF5.8* directly activates the expression of *LcTPSbms*.** (a) The yeast one hybrid (Y1H) analysis of *LcDOF5.8* binding to the promoter of *LcTPSbms*. No basal activities of *TPSbms* promoter were detected in yeast grown on SD medium lacking Ura in the presence of 200 ng/mL aureobasidin A (AbA). The interaction was evaluated based on the growth conditions of transformed yeast on SD medium lacking Leu in the presence of 200 ng/mL AbA. (b) Subcellular localization of *LcDOF5.8* in tobacco leaves. The nucleus marker (NLS-mCherry) was used as the positive control. The merged images indicate the co-localization of GFP and mCherry signals. Scale bars are 20 µm. (c) Electrophoretic mobility shift assays (EMSAs) showing the binding ability of *LcDOF5.8* to the promoter of *LcTPSbms in vitro*. Shifted bands, indicating the generation of DNA-protein complexes, are marked by red arrows. ‘+’ and ‘-’ indicate the presence and absence, respectively. ‘++’ denotes increased amount of mutant or unlabeled probes used for binding competition. (d) Transcription activity of *LcDOF5.8* in tobacco cells. The transcription activity of *LcDOF5.8* was assessed using the dual-luciferase reporter assay by detecting the ratio of LUC to REN. (e) *LcDOF5.8* enhanced the expression of *LcTPSbms in vivo*. The effector and reporter vectors were co-transformed into tobacco leaves. After incubation for 48–72 h, the ratio of LUC to REN was detected. Error bars indicate SDs from at least six replicates. Asterisks indicate a significant difference (Student’s *t*-test: ** *p* < 0.01).

To validate the interaction between LcDOF5.8 and the promoter of *LcTPSbms*, we cloned the coding sequence of *LcDOF5.8* into the pGADT7 vector and the promoter region of *LcTPSbms* into the pABAi vector. Subsequently, these constructs were co-transformed into yeast cells, and their growth was assessed on SD/-Leu medium supplemented with Aureobasidin A (AbA). To illustrate (Fig. 6a), robust yeast growth was observed only in the presence of both LcDOF5.8 and the *LcTPSbms* promoter, indicating that LcDOF5.8 specifically binds to the *LcTPSbms* promoter, thereby activating the reporter genes in yeast. Furthermore, subcellular localization experiments confirmed that LcDOF5.8 is localized within the nucleus (Fig. 6b), reinforcing its role as a functional transcription factor.

To further corroborate the specific binding of LcDOF5.8 to the promoter of *LcTPSbms*, we conducted an electrophoretic mobility shift assay (EMSA). For illustration (Fig. 6c), the recombinant LcDOF5.8 protein exhibited a clear binding affinity towards the labeled probes containing the TACTTTAT motif derived from the *LcTPSbms* promoter (−1282 to −1225 bp). Notably, this binding was specifically abrogated when unlabeled probes of the identical sequence were introduced, whereas mutated probes had no such effect, conclusively demonstrating the specific recognition and interaction between LcDOF5.8 and the DOF motif present within the promoter region of *LcTPSbms*.

To evaluate the transcriptional regulatory potential of LcDOF5.8 on *LcTPSbms*, we initially employed the dual luciferase reporter system (dual-LUC) as a platform to examine its transcriptional activity. Our results demonstrated that transient expression of pBD-LcDOF5.8 in tobacco leaves led to a marked upregulation of the LUC reporter (Fig. 6d), indicating that LcDOF5.8 acts as a transcriptional activator. To substantiate this finding and investigate whether LcDOF5.8 could stimulate the expression of *LcTPSbms* specifically, we utilized the dual-LUC system again. By co-expressing the effector construct pGreenII 62-SK-LcDOF5.8 with the *Pro_LcTPSbms_*-LUC reporter, we observed a significant enhancement in the LUC/REN ratio compared to the control (Fig. 6e). This observation conclusively proves that LcDOF5.8 is indeed capable of activating the promoter activity of *LcTPSbms*, thereby driving its transcription.

### 3.6 Transient overexpression of *LcDOF5.8* in litchi aril significantly increases α-bergamotene content

**Fig. 7:**
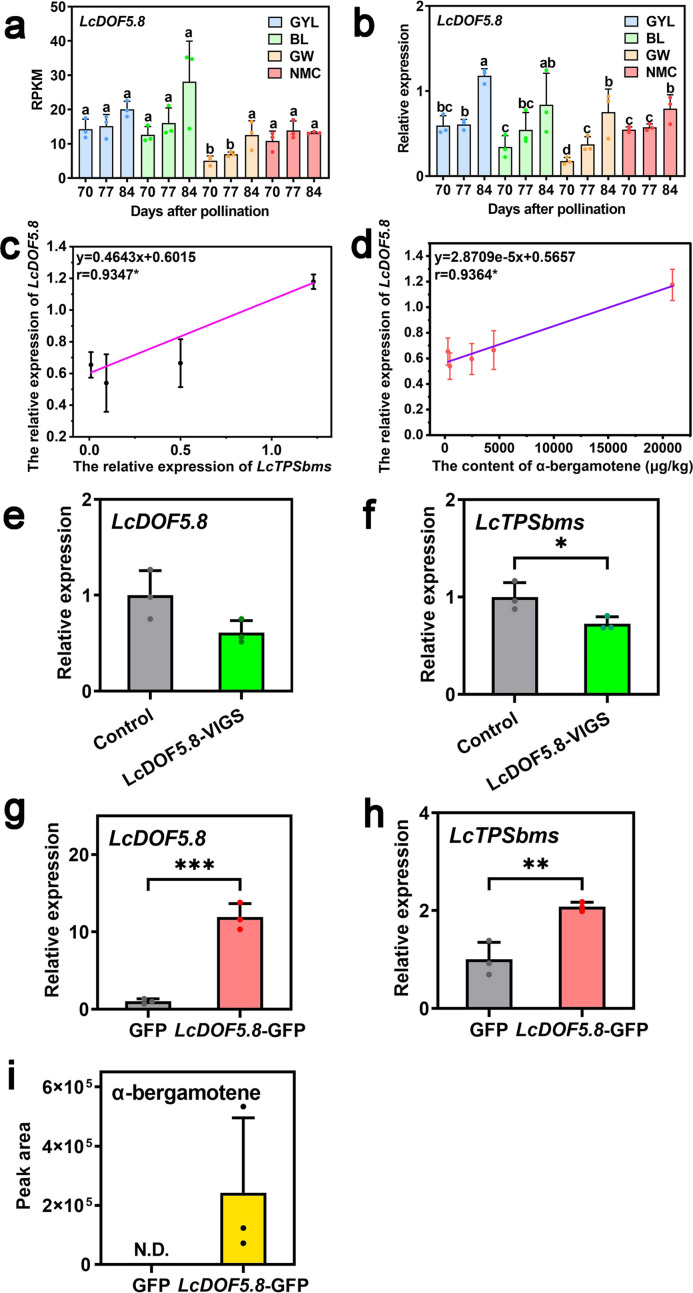
**Overespression of *LcDOF5.8* in litchi aril significantly increases the α-bergamotene content.** The expression patterns of *LcDOF5.8* at 70, 77, and 84 DAP in ‘GYL’, ‘BL’, ‘GW’, and ‘NMC’ litchi cultivars under RNA-seq (a) and qRT-PCR (b) analysis. RPKM, Reads Per Kilo base per Million reads. Correlations between the expression of *LcDOF5.8* and the expression of *LcTPSbms* (c), and the content of α-bergamotene (d) at 84 DAP across ‘GYL’, ‘BL’, ‘GW’, and ‘NMC’ litchi cultivars. (e) The expression of *LcDOF5.8* was down-regulated in *LcDOF5.8*-silenced aril. (f) The expression of *LcTPSbms* was significantly decreased in *LcDOF5.8*-silenced aril. The expression of *LcDOF5.8* (g) and *LcTPSbms* (h), and the content of α-bergamotene (i) was obviously enhanced in *LcDOF5.8*-overexpressed litchi aril. The error bars indicate mean ± SD. Different letters indicate significant pairwise differences according to the Duncan’s test (*p* < 0.05). Asterisks indicate a significant difference (Student’s *t*-test: * *p* < 0.05, ** *p* < 0.01). N.D., not detected. At least three independent experiments were performed.

To investigate the potential involvement of LcDOF5.8 in regulating α-bergamotene synthesis, we initially analyzed its expression patterns across four litchi varieties at 70, 77, and 84 DAP. According to the RNA-seq results (Fig. 7a), *LcDOF5.8* exhibits an overall upward trend in the fruit ripening process across four cultivars. To validate this finding, qRT-PCR was performed. As depicted in Fig. 7b, the qRT-PCR results concurred with the RNA-seq data, demonstrating a progressively enhanced expression of *LcDOF5.8* during the three ripening stages in all four cultivars. Notably, *LcDOF5.8* showed the predominant expression in ‘GYL’, closely followed by ‘BL’, while relatively lower expressions were observed in ‘GW’ and ‘NMC’, especially at the fruit full maturation stage (84 DAP). Furthermore, a positive correlation was found between *LcDOF5.8* expression and the expression of *LcTPSbms* (Fig. 7c). Furthermore, among the six DOFs, LcDOF5.8 displayed the most significant positive correlation with the expression patterns of *LcTPSbms* based on the correlation analysis results (Figs. 7c and S6). Notably, we also observed a strong congruence between the expression pattern of *LcDOF5.8* and the content distribution of α-bergamotene in four litchi cultivars (*r* = 0.94) (Fig. 7d). These findings suggest a plausible regulatory role of LcDOF5.8 in the synthesis of α-bergamotene.

To further elucidate the role of LcDOF5.8 in modulating α-bergamotene synthesis, a series of experiments was conducted utilizing ‘NMC’ aril as the test material. First, we utilized virus-induced gene silencing (VIGS) in the ‘NMC’ litchi aril. Following a week of transformation, a decrease in *LcDOF5.8* expression was observed in the silenced aril compared to the control (Fig. 7e). Significantly, the expression of *LcTPSbms* was significantly suppressed in the *LcDOF5.8*-silenced litchi aril (Fig. 7f). Moreover, we transiently overexpressed *35S:LcDOF5.8-GFP* and the *35S:GFP* control in ‘NMC’ aril, respectively. Upon comparison with the control, we observed a marked upsurge in *LcDOF5.8* expression within the *LcDOF5.8*-overexpressed litchi aril, indicating the successful transformation (Fig. 7g). Remarkably, the expression of *LcTPSbms* was significantly induced in the *LcDOF5.8*-overexpressed litchi aril (Fig. 7h), reinforcing the regulatory mechanism of *LcDOF5.8* on *LcTPSbms*. Additionally, we quantified the α-bergamotene content in the *LcDOF5.8*-overexpressed litchi aril. As vividly illustrated in Fig. 7i, a substantial increase in α-bergamotene content was observed within the *LcDOF5.8*-overexpressed litchi aril in comparison to the control. In conclusion, these findings collectively suggest that LcDOF5.8 could facilitate the biosynthesis of α-bergamotene by directly upregulating the expression of *LcTPSbms* in the litchi aril.

## 4 Discussion

Fruit aroma is the pivotal characteristic of horticultural plants and the key indicator for evaluating fruit quality. The unique content and ratios of volatile compounds are what give rise to the distinct aroma that is typical of different fruit varieties [[1], [2], [3], [4]]. Here, we report a novel regulatory module consisting of LcDOF5.8 and LcTPSbms that plays a key role in the biosynthesis of α-bergamotene in the aril, the edible part of the litchi fruit. This regulatory module is particularly interesting because α-bergamotene, a type of sesquiterpene, is a significant contributor to the overall aroma of the fruit. Our findings not only shed light on the molecular mechanisms behind the production of this important aroma compound but also potentially open up new avenues for improving the flavor profile of litchi and possibly other fruits as well.

Sesquiterpenes, as a significant contributor to fruit aroma, constitute the largest subgroup of terpenes and have found widespread applications in the flavor, fragrance, pharmaceutical, and biofuels industries [10,11]. Among the myriad of sesquiterpenes, α-bergamotene is a particularly important compound, renowned for its sweet and citrusy aroma that greatly enhances the characteristic aroma of various fruits [30,[55], [56], [57], [58], [59], [60], [61], [62], [63]]. The distinct content of α-bergamotene imparts a unique aroma to plants. For instance, a study on 10 potato varieties identified the Mila variety as distinct due to its high level of α-bergamotene, β-bergamotene, β-farnesene, and other sesquiterpenes in leaves [64]. Similarly, α-bergamotene and β-bisabolene have been identified as important chemical markers to differentiate citron, lemon, and lime from other citrus species [57,61]. In our current research, we observed a ladder-like content distribution of α-bergamotene across three different fruit developmental stages in four distinct litchi cultivars, suggesting that the varying content of α-bergamotene might contribute to the formation of unique aroma characteristics in different litchi cultivars. Our findings underscore the potential for manipulating α-bergamotene levels as a means to enhance or modify the aroma of litchi and other fruits, offering exciting prospects for the horticultural and flavor industries.

Terpene synthases (TPSs) are the key enzymes responsible for the production of terpenes in plants [26]. Specifically, the synthesis of bergamotene, a valuable sesquiterpene known for its sweet and citrusy aroma, has been documented to be mediated by specific *TPS* genes, such as *LoTPS7* in sweet pea [32], *TPS10* in maize [33], *SaSSy* in sandalwood [34], and *SbTPS3* and *SbTPS5* in sorghum [35]. Although α-bergamotene has been previously detected in litchi fruit [43], the specific TPS genes involved in its biosynthesis in this context were not well understood. Our study revealed a close correlation between the expression of *LcTPSbms* and the content distribution of α-bergamotene in three fruit ripening stages across four different litchi cultivars, suggesting LcTPSbms may play a key role in the formation of α-bergamotene (Fig. 2). To validate this hypothesis, we conducted *in vitro* and *in vivo* catalytic experiments, which demonstrated a predominant production of α-bergamotene formed by LcTPSbms (Figs. 3, 4). Terpenoid synthase genes are known for their multifunctionality and functional redundancy, enabling them to catalyze the synthesis of a diverse array of compounds. In our study, we revealed that LcTPSbms can also synthesize β-farnesene, a compound characterized by its fresh, green, and fruity aroma. β-farnesene is commonly found in plant essential oils, such as those from sweet orange, rose, and orange oils [[65], [66], [67]]. However, a correlation analysis between *LcTPSbms* expression and β-farnesene content showed only a weak correlation (Fig. S3). This suggests that other terpenoid synthase genes in litchi may be primarily responsible for β-farnesene production.

Previous studies have revealed that the expression of *TPS* genes is under the transcriptional control of multiple families of transcription factors (TFs) [36]. In the present study, 85 candidate TFs that are closely associated with the expression of *LcTPSbms* were detected using WGCNA analysis, mainly including AP2/ERF, MYB, C2C2-Dof, Trihelix, and Homebox families (Fig. 5). By integrating the results from WGCNA analysis, promoter region analysis and yeast one-hybrid experiments, we identified the DOF transcription factor (LITCHI011105), designated as LcDOF5.8, as a potential upstream regulator of *LcTPSbms.* The DOF TFs have been shown to participate in diverse processes including hormone signaling, responses to abiotic stress, light perception, and carbon and nitrogen metabolism [41]. However, their specific function and transcriptional regulatory mechanisms in terpene biosynthesis have not been extensively explored. In this study, *LcDOF5.8* exhibits a strong correlation with *LcTPSbms* expression and α-bergamotene content across four litchi cultivars (Fig. 7a-d). Through a series of experiments, including EMSA, dual-luciferase reporter assay, and VIGS, we demonstrated that LcDOF5.8 can directly bind to the *LcTPSbms* promoter, thereby activating its expression (Figs. 6, 7e, 7f). Additionally, we also analyzed the expression patterns of five other DOF TFs identified by WGCNA. However, only *LcDOF5.8* exhibited high expression in the aril (Fig. S4) and showed a positive correlation with the expression pattern of *LcTPSbms*. Transient overexpression of *LcDOF5.8* in litchi aril led to the upregulated expression of *LcTPSbms* and consequently increased α-bergamotene content compared to control (Fig. 7g-7i). Collectively, we regard LcDOF5.8 as the key regulator of *LcTPSbms*. This finding represents the first evidence of DOF TFs positively regulating biosynthesis of terpenes through the direct promotion of *TPS* gene expression, highlighting the significant regulatory role of LcDOF5.8 in the production of α-bergamotene in litchi fruit.

Although DOF TFs play a crucial role during plant growth and development, there is a scarcity of reports on the molecular mechanisms regulating DOF TFs. In the present study, the expression of *LcDOF5.8* showed an upward trend during the fruit ripening process in litchi (Fig. 7a, 7b), which was highly governed by plant hormones. Notably, abscisic acid (ABA), a sesquiterpenoid phytohormone [68], has been shown to modulate ripening-related gene expression, suggesting a potential role in the upregulation of *LcDOF5.8*. Previously studies have proved the application of ABA could regulate the aroma of grape berries [69], kiwifruit [70], tomato [71], and apple [63]. Interestingly, motifs responding to ABA signaling were found in the promoter of *LcDOF5.8* (Dataset S1). These results indicated the possibility that *LcDOF5.8* might be regulated by ABA to affect the fruit aroma. Furthermore, *LcDOF5.8* showed the deferential expression pattern across four different litchi cultivars, which may correlate with the varying content of carbohydrates, such as glucose, fructose, and sucrose, produced by photosynthesis. Previous studies have demonstrated that sugars (glucose, fructose, and sucrose) can affect the fruit aroma in tomato [72,73], pear [62], kiwifruit [74], and mango [75]. Notably, we also found core light response elements in the promoter of *LcDOF5.8* (Dataset S1), implying that *LcDOF5.8* might also be regulated by sugar levels formed through the photosynthetic pathway, and then affect volatile compounds in litchi fruit. These potential molecular mechanisms warrant further in-depth investigation to fully elucidate the complex interplay between hormonal signaling, carbohydrate metabolism, and the regulation of aroma compound biosynthesis in fruit.

In summary, our research uncovered a novel regulatory mechanism whereby LcDOF5.8 controls the production of α-bergamotene by directly activating *LcTPSbms* expression. We observed that the distinct content distribution patterns of α-bergamotene have a strong correlation with the expression of *LcTPSbms* across four litchi cultivars during three fruit developmental stages. Notably, LcDOF5.8 significantly upregulates the expression of *LcTPSbms*, mirroring its expression pattern. Besides, transient overexpression of *LcDOF5.8* in litchi aril led to an enhanced expression of *LcTPSbms* and subsequently increased production of α-bergamotene. These findings have shed light on a previously unrecognized function of DOF transcription factors in the formation of terpenes and have delineated a molecular regulatory pathway that could be harnessed to enhance fruit aroma and guide litchi breeding programs in the future. By manipulating the expression of *LcDOF5.8* and its target gene *LcTPSbms*, we may be able to develop litchi cultivars with improved aroma profiles, thereby increasing their market appeal and consumer satisfaction. This research not only advances our understanding of the genetic basis of fruit aroma but also provides a foundation for innovative breeding strategies aimed at enhancing the sensory qualities of horticultural crops.

## CRediT authorship contribution statement

**Yimeng Wang:** Writing – original draft, Project administration, Investigation. **Zhuoyi Liu:** Data curation. **Minghui Wang:** Methodology. **Dawei Qian:** Methodology. **Ke Ma:** Resources, Investigation. **Minglei Zhao:** Writing – review & editing, Supervision. **Jianguo Li:** Writing – review & editing, Funding acquisition. **Xingshuai Ma:** Writing – review & editing, Supervision.

## Declaration of competing interest

The authors declare that they have no conflicts of interest in this work.

## Appendix Supplementary materials

Image, application 1

Image, application 2
