# Supplementary material for: Regulation of α-bergamotene biosynthesis by the LcDOF5.8-LcTPSbms regulatory module in litchi fruit
Source: Fundam Res. 2025 Dec 17;6(4):2262–72. doi: 10.1016/j.fmre.2025.12.004 (PMC13424721; doi:10.1016/j.fmre.2025.12.004)
Supplement: Supplementary file 1 [file mmc1.doc]

**Fig. S1. Multiple alignments of LcTPSbms protein with TPS proteins from sweet pea [32], maize [33], sandalwood [34], and sorghum [35], which have been proved to play a role in the biosynthesis of α-bergamotene.**

**
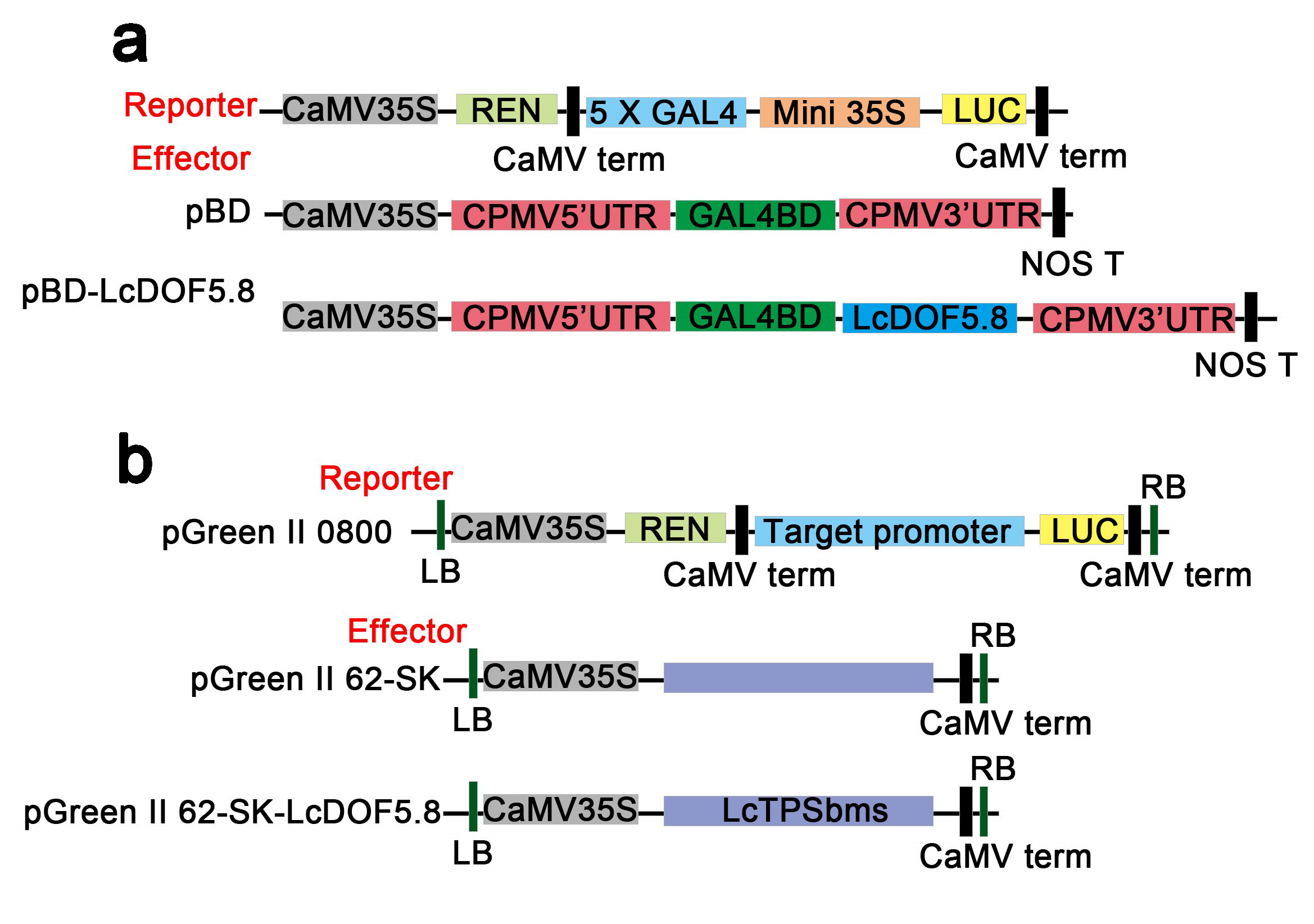
**

**Fig. S2. Schematic maps of constructs used in this study.** (a) Diagram of reporter and effector constructs used in the transcription activity analysis of LcDOF5.8. (b) Diagram of reporter and effector constructs used in the transient assays of LcDOF5.8, which activate the expression of promoter of *LcTPSbms*.

**
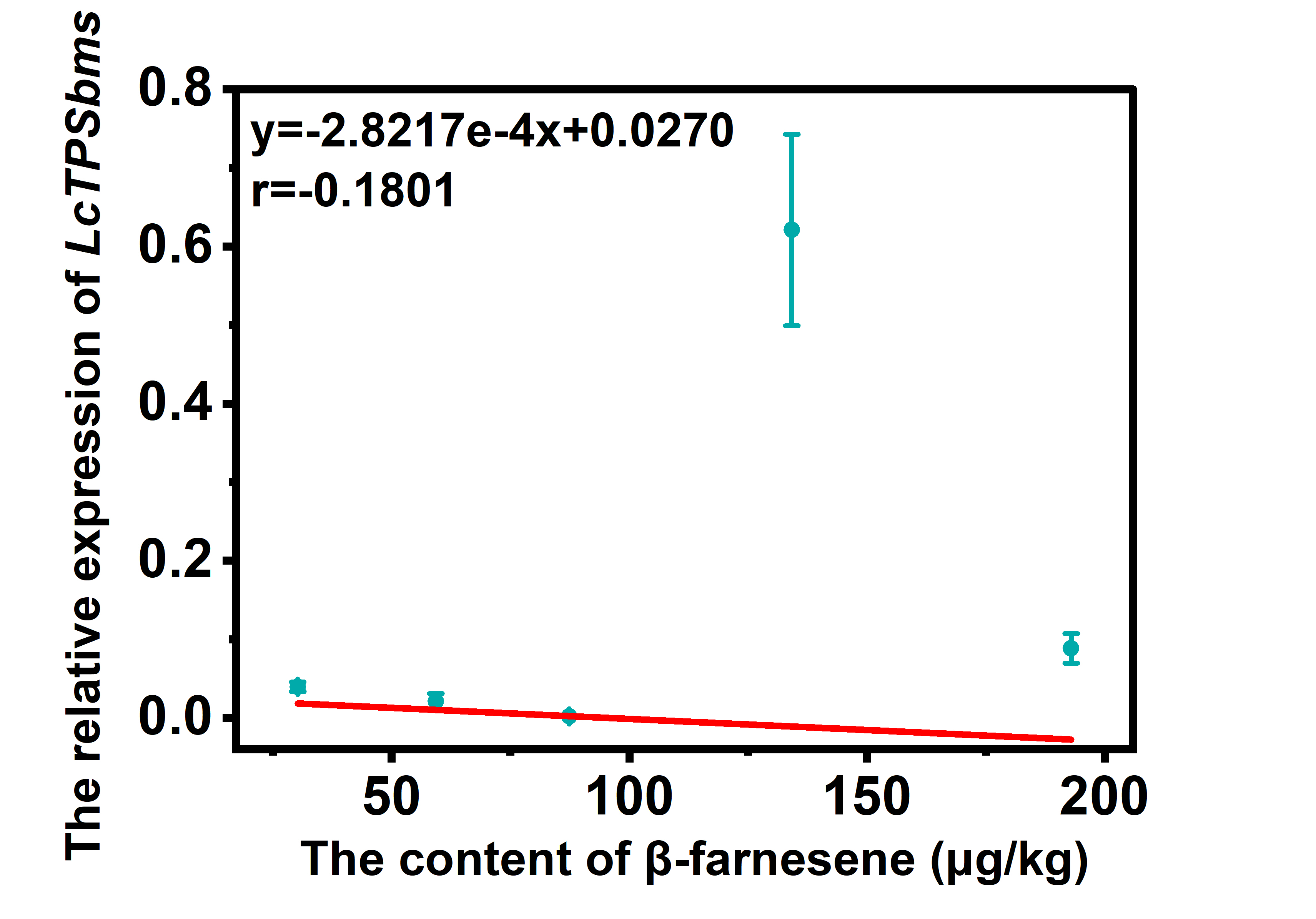
**

**Fig. S3. Correlations between the expression of *LcTPSbms* and the content of β-farnesene at 84 DAP in** **‘GYL’, ‘BL’, ‘GW’, and ‘NMC’ litchi cultivars.**

**
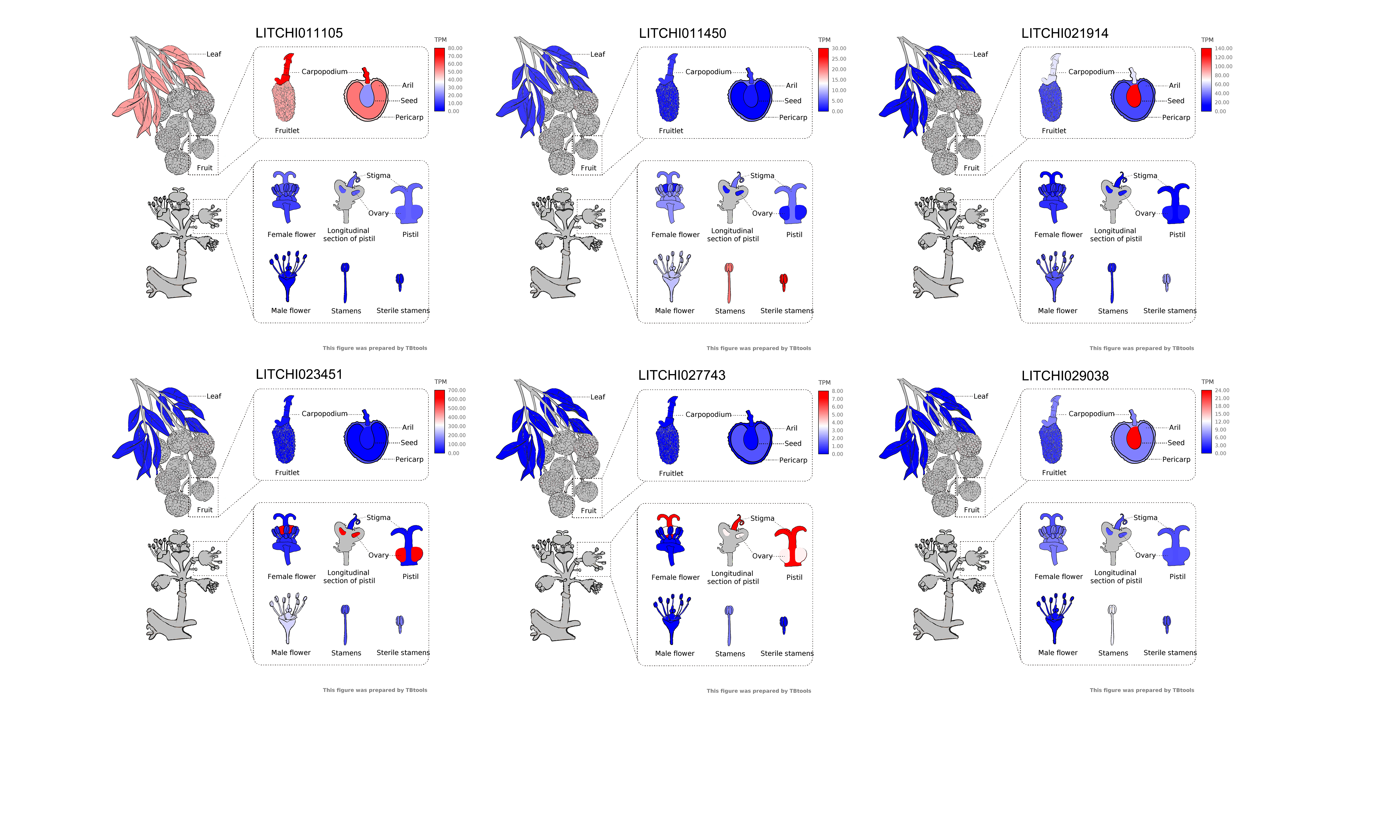
**

**Fig.** **S4. Tissue-Specific expression results of the six DOF transcription factors. The result is sourced from the Sapindaceae genome database(http://www.sapindaceae.com/index.html) [36].**


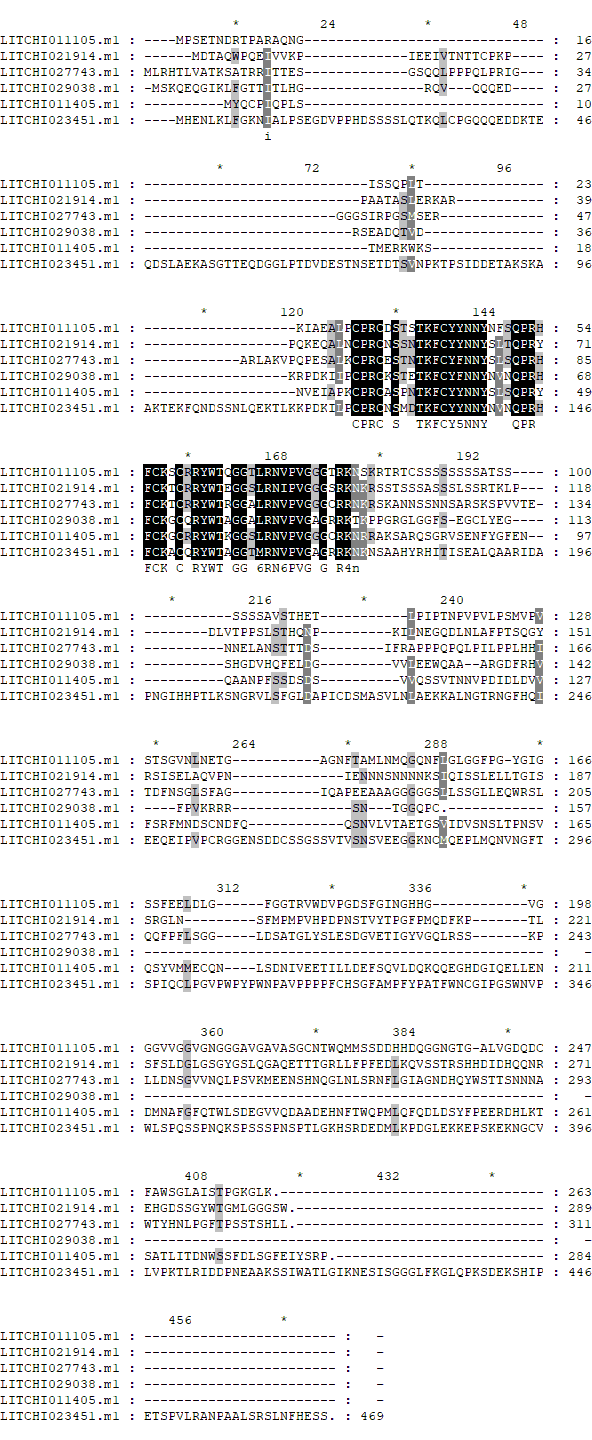


**Fig. S5. Multiple sequence alignmennt of six DOF protiens.**

**

**

**Fig. S6. Correlations between the expression of *LcTPSbms* and the other five DOF transcription factors at 84 DAP in ‘GYL’, ‘BL’, ‘GW’, and ‘NMC’ litchi cultivars.**

**
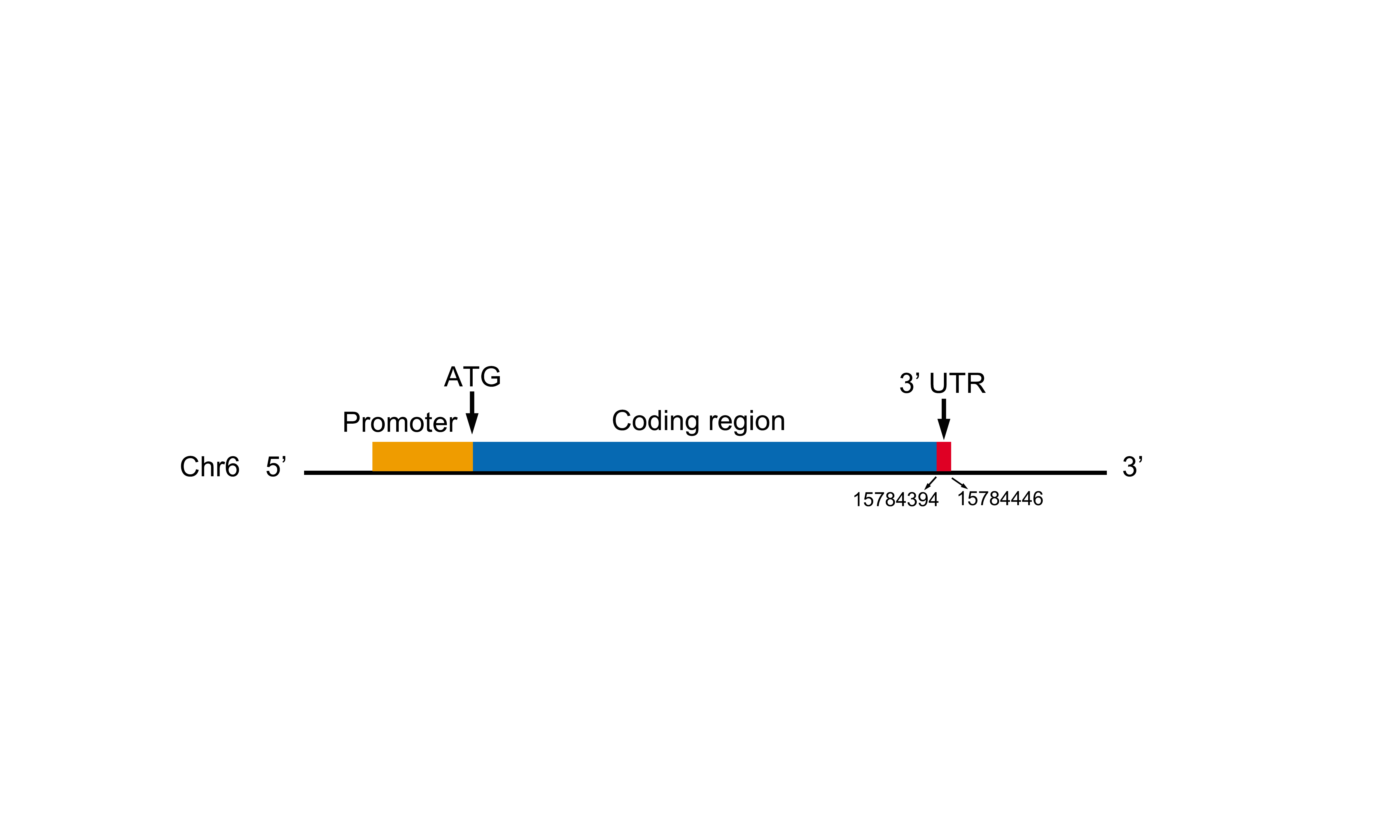
**

**Fig. S7. The promoter, coding region, and 3' UTR region of *LcTPSbms* are located on chromosome 6 using data from the Sapindaceae genome database (http://www.sapindaceae.com/index.html) [36].**


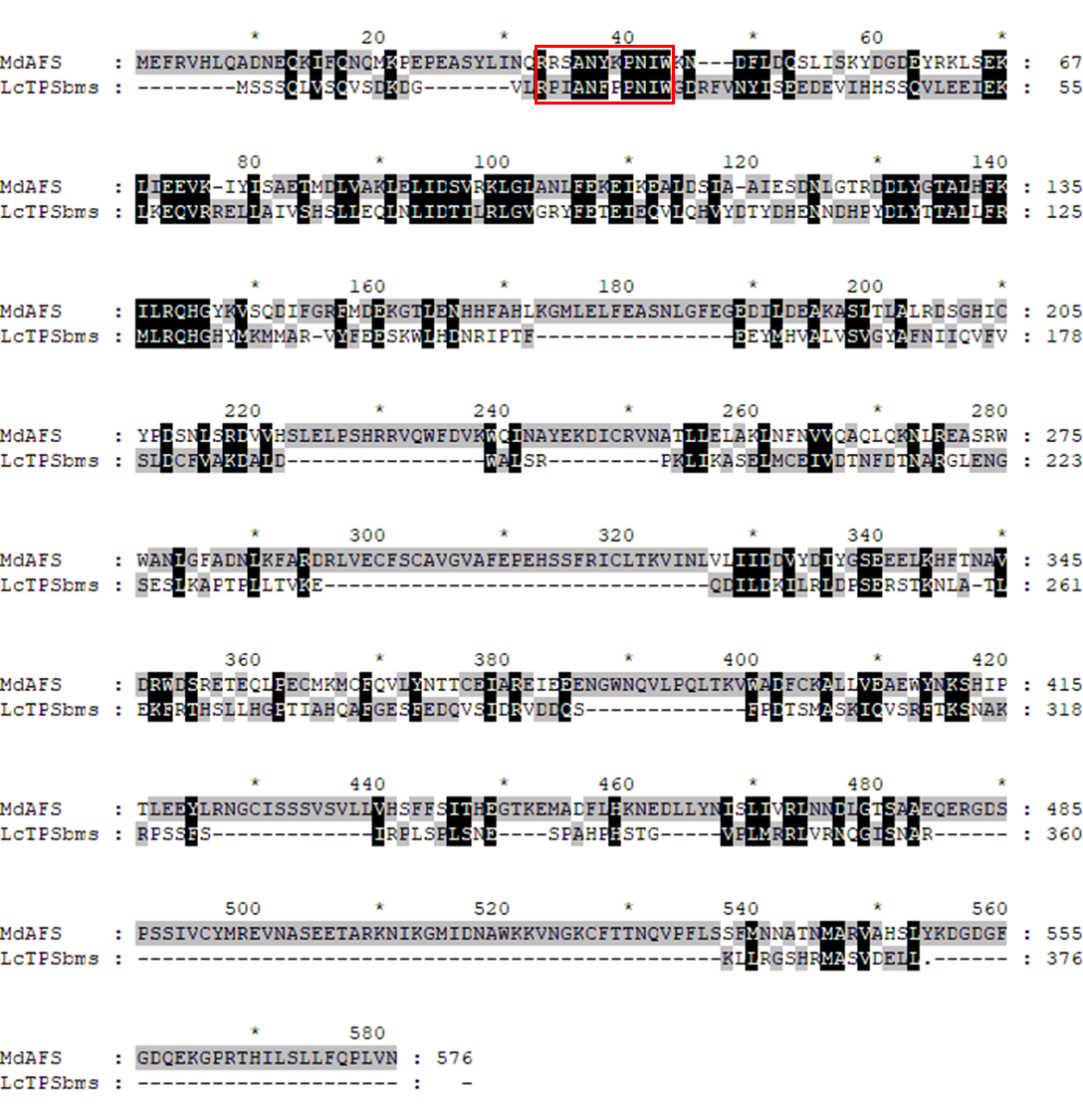


**Fig. S8. Sequence analysis of *LcTPSbms* and *MdAFS.***
