# Supplementary material for: Regulation of α-bergamotene biosynthesis by the LcDOF5.8-LcTPSbms regulatory module in litchi fruit
Source: Fundam Res. 2025 Dec 17;6(4):2262–72. doi: 10.1016/j.fmre.2025.12.004 (PMC13424721; doi:10.1016/j.fmre.2025.12.004)
Supplement: Supplementary file 2 [file mmc2.doc]

**Table S1 Summary of primers used in this study.**

| **Assay** | **Primer sequence (5’-3’)** | **Restriction Site** | |
| --- | --- | --- | --- |
| Subcellular localization | *LcTPSbms-GFP-F*: caaattcgcgaccggtATGTCTAGCAGCCAATTAGTTTCA  *LcTPSbms-GFP-R*: tgctagtcataccggtTATAGGAACAGGATCTATGAGC  *LcDOF5.8-GFP-F:*caaattcgcgaccggtATGCCATCGGAAACCAACGA  *LcDOF5.8-GFP-R:*tgctagtcataccggtCTTCAAGCCCTTCCCTGGTG | *Age* Ⅰ | |
| qRT-PCR | *LcDOF5.8-qF:* GGTGGGTTTCCTGGGTATGG  *LcDOF5.8-qR:* TTACCGACACCACCCACAAC  *LcTPSbms-qF*: GCGTTGGCATCAGTTGGTTT  *LcTPSbms-qR*: ACTCAAGGCCCAATCTAAGGC  *LcEF-1α-qF*: CGTGGATTCGTCGCATCTAAC  *LcEF-1α-qR*: CCGCCTGTCAATCTTGGTCAT |  | |
| Virus-induced gene silencing | *LcDOF5.8-pTRV2-F:*gcctccatggggatccGACCGTACACCGGCTCGAGC  *LcDOF5.8-pTRV2-R:* cttcgggacatgcccgggAGCGGAGGAAGAAGACGAAG | *Bam*HI  *Sma*I | |
| EMSA assay | *pMAL-c2-LcDOF5.8-F*: agggaaggatttcagaattcATGCCATCGGAAACCAACGA  *pMAL-c2-LcDOF5.8-R*: actctagaggatccgaattcCTACTTCAAGCCCTTCCCTG  *LcTPSbms-probe-F*: ATCATAATTTAACTTTTATTTTTTAtactttatTTGTTCTCTTCGAACTAATTAAAGA  *LcTPSbms-probe-R*: TCTTTAATTAGTTCGAAGAGAACAAataaagtaTAAAAAATAAAAGTTAAATTATGAT  *LcTPSbms-mprobe-F*: ATCATAATTTAACTTTTATTTTTTAcgtcccgcTTGTTCTCTTCGAACTAATTAAAGA  *LcTPSbms-mprobe-R*: TCTTTAATTAGTTCGAAGAGAACAAgcgggacgTAAAAAATAAAAGTTAAATTATGAT | | *Eco*R I |
| Dual-luciferase transient expression assay | *LcDOF5.8-pBD-F*: tcgccgaccggtaggcct ATGCCATCGGAAACCAACGA  *LcDOF5.8-pBD-R*: aaccagagttaaaggcctCTTCAAGCCCTTCCCTGGTG  *LcDOF5.8-pGreenII 62-SK-F*: tagaactagtggatccCTTCAAGCCCTTCCCTGGTG  *LcDOF5.8-pGreenII 62-SK-R*: cggtatcgataagcttCTACTTCAAGCCCTTCCCTG  *Pro_LcTPSbms_-LUC-F:* tatagggcgaattggTTTTATTCATATTTATCTACTA  *Pro_LcTPSbms_-LUC-R:* ttggcgtcttccatggATTTGATGGCTGCTTAAAAATA | *Stu* Ⅰ  *Bam*HI  *Hin*dIII  *Kpn* Ⅰ  *Nco* Ⅰ | |

**Dataset S1 Nucleotide sequence of *LcTPSbms* promoter.** *cis*-acting elements involved in light responsiveness is indicated in yellow. *cis*-acting elements involved in the ABA responsiveness is indicated in cyan. Translation start site (ATG) is shown in red.

TTTTATTCATATTTATCTACTACTTGTTATATCTCTATTATGATAATAATGATATATATCATAAAAAATAAATGTAAATGTATTTATAATTTTCATTGAAATATTAATATCTTATTACACATTTTAAATATTGATATCTCTAGCTTAATTTGAAAATCAAATCAATAAATCCGAAAATTTTACATTTTAATTTGTTATATATAATATCTCAATGTACAATCATAATTTAACTTTTATTTTTTATACTTTATTTGTTCTCTTCGAACTAATTAAAGAGATATATTATGCTATATTTGTTTAAGTATAATTTCTAACAAAAACTTAATTTTTAATTTGAATGTGAAATTTACATAATTTGATTGTTAATGGTTATAATATAATCACAAATTAATAATTAAGTTATCTTTCTTTTATACAAATTATCAATTGATAAAAATATTATTTCATTTTTTCTTATACGTGAAAATTTGAGATTTTTAAAATGTAAAATTATGTGTACTTAAATGTAGTATTTATTTTCATTTTTAGTTATTCTGGGTTGTTGGAGATAACTAAAAATTAAATGAACTGTAAATTTCATATTTTACGATTTAAGTTTTAGTTATTTTTAATATCATTATTGATTTATTAAAATGTCTCATCTAATATAAATTTATCCCCCTTCGTTTTCAACGGATTTGTCTCACTTCATACTATTATTAAACTACTAGACAAACCACTAGCCTTGTAGCAATTACAGACAATTATTCGAAAATTATAGACAATTATTTGATAAGATAGATATTTTAACTGATGACGACTACAAACAATTATTAGATAAGATAGATATTTTAACTGATAGCTAGATATTTTTATGAGAAATTATACAGATAGGAGGACAGATATTTTCAGACAAACTACACATTATCATAGACATTTTACCAAACTATCGCGGGTGGTTCTAATGTAATCATAAATCAATAATTAAGTCATCTTTATTTTATATGAATTATTATTTGATAAAAATGTTATTTCATTTTTTTATACCTTAAAATTTGAAAATTTTAAATTGTCAAATTATATATATACTTAAATGTAGTATTTATTTTCATTTTAGTTATTTTAGATTAATGGAGATAACTAGAAATTAAATAAAATGTAAATTTCATATTTTGCCAATAAATTTTAGTTATTTTTAATAATATTATTAATTTATTAAAATATCCCATCTAATATGAATTTTTAGATATGTCACTCACATAATAATATATTAAGATTAGACTTAAAGCCTCAAGTAAGAAGTAAAATAATATTATTTGATTATCTCAAGAAAGCGAAATACATATAATCAAATTAACGGCAATATATATATATATATATAATGATTTTACGATCAGTGTCTCCTAACTTTAAAAGGTTATAATAAATTAATTAAATTCATCTATTTTCAACGTAATATGATACGCGATAAATACTGATTCTCACATAACCATTTTGCTTATTTTTAAGCAGCCATCAAATATG
